# A comparison of the existing recommendations for human and veterinary clinicians on the management and prevention of the zoonotic aspects of dermatophytosis: A scoping review

**DOI:** 10.1371/journal.pone.0344010

**Published:** 2026-03-12

**Authors:** Caroline O’Connor, Daisy Hollister, Richard Barlow, Hannah Wainman, Alison Ashmore, Lisa Morrow, Jenny Stavisky, Kathryn Griffiths, Christina Kuhl, Marnie L. Brennan

**Affiliations:** 1 School of Veterinary Medicine and Science, University of Nottingham, United Kingdom; 2 Queen Elizabeth Hospital, Birmingham, United Kingdom; 3 Centre for Applied Excellence in Skin and Allergy Research (CAESAR), University of Bristol Bristol, United Kingdom; 4 University Hospitals Bristol and Weston NHS Foundation Trust, Bristol Dermatology Centre, Bristol Royal Infirmary, Bristol, United Kingdom; 5 University of Nottingham Libraries, The University of Nottingham, United Kingdom; 6 VetPartners Head Office, York, United Kingdom; Universidade Federal de Minas Gerais, BRAZIL

## Abstract

**Background:**

Dermatophytosis (or ringworm) is a highly infectious zoonotic disease commonly found in humans and multiple animal species globally. With children under 5 years of age being the most at risk human patient group, and dermatophytosis the zoonosis most frequently contracted by veterinary professionals in the UK from their patients, it is of significance to both human and animal patients. Little is known as to whether there is recognition in both human medical and veterinary guidance of the importance of their opposite clinical counterpart. In addition, it is unknown whether the recommendations for zoonotic disease management and prevention are complementary between the two disciplines.

**Objectives:**

The aim of this scoping review was to assess all human medical and veterinary guidelines pertaining to zoonotic dermatophytosis, to explore how the zoonotic aspects of the disease were reported and to determine if there was conflicting or complementary advice between the two disciplines on disease management and prevention. Sources of evidence: Medline, CAB Abstracts, and Embase were searched for relevant literature and the results assessed and filtered using inclusion and exclusion criteria. A targeted grey literature search was also performed.

**Eligibility criteria:**

To be included, broadly all publications had to mention dermatophytes or specifically named dermatophyte species, and mention ‘guidelines’ or ‘protocols’. Publications needed to also mention terms relating to ‘zoonoses’; for veterinary publications, be focused on cattle, dogs and cats and for human medical publications, mention the clinical manifestation of dermatophytosis (e.g. tinea capitis). Some mention of risk factors for zoonotic transmission needed to also be included.

**Charting methods:**

A data charting form was used to extract data pertaining to dermatophyte species discussed, animal species discussed, prevalence and risk factors, zoonotic risk factors, zoonotic recommendations for humans, transmission, diagnostic testing, treatment, monitoring response to treatment, and prevention and management from the included studies. A critical appraisal process using the AGREE II tool was conducted to identify the common limitations of the shortlisted published papers.

**Results:**

Of the 554 human and 137 veterinary publications screened, 5 of each publication source met the inclusion criteria. Although data were charted across several variables, none of the publications used an evidence-based guideline approach in their construction (e.g. GRADE, AGREE processes) and a significant proportion of papers provided limited supporting evidence for their recommendations. There were significant gaps and minimal synergies between veterinary and human medicine recommendations. The human literature had limited information pertaining to zoonotic recommendations.

**Discussion:**

A minimal number of studies have been conducted regarding zoonotic dermatophytosis, both in human medical and veterinary disciplines. There is a lack of good quality, detailed information about the prevention and management of the zoonotic aspects of the disease, indicating that there is a need for the development of evidence-based guidelines to support human and veterinary clinicians making decisions about these patients.

**Conclusion:**

Evidence-based guidelines, inclusive of high quality information pertaining to the zoonotic aspects of the disease for both humans and animals, should be generated. Ideally human and veterinary representatives would work together to generate cohesive and complementary guidance.

## 1. Introduction

### 1.1. Rationale for the review – dermatophytosis infection in animals and humans

Dermatophytosis, colloquially referred to as ringworm, is a superficial fungal skin infection that is highly prevalent in both humans and other animals worldwide [1–5]. Although not a fatal disease, proper diagnostic, treatment, and management protocols are essential in order to prevent more serious complications, particularly for at-risk groups [5,6]. Transmission between other animals and humans is possible and due to its highly contagious and infectious nature, dermatophytosis is regarded as one of the most common dermatological zoonotic diseases [7–9]. However, an accurate transmission rate between animals and humans is unknown [1].

There are a wide range of potential sources of zoonotic infection. Dermatophytosis commonly affects cattle and horses and also occur in other species (e.g., guinea pigs, rabbits, rodents, and hedgehogs, to name a few). However, because of their frequency of contact with humans, cats and dogs have commonly been identified as sources of infection; *Microsporum canis* is the zoophilic dermatophyte most commonly responsible [4,5,8]. *Trichophyton mentagrophytes* (including *Arthroderma vanbreuseghemii*) has been named as another common cause of canine dermatophytosis, and bovine infection is most frequently caused by *Trichophyton verrucosum* [1,10,11].

Diagnosis and treatment in animals is challenging, and infection occurs commonly in environments such as animal shelters where animals are housed in close contact in combination with a lack of appropriate prevention and control protocols which can lead to widespread infection [1,5]. In addition to shelter outbreaks, dermatophytosis is frequently seen on farms, especially those with cattle [2,11]. In situations where livestock are in close contact, dermatophytes can transmit directly between animals (as well as to farm workers) and will also shed spores that will contaminate the environment and sustain herd infection for several years [9,12]. Several sources report that younger animals are more likely to contract infection [4,13], with Bond [14] and Segal and Elad [9] suggested a less-mature immune system could be a risk factor. Many sources also name physiological stress, poor hygiene, and immunosuppression as predisposing factors for the development of clinical disease [1,2,4,8,15].

Tinea pedis, cruris, and capitis are the three most common manifestations of dermatophytosis in humans [16]. *Microsporum canis* is the most common zoophilic cause of Tinea capitis cases in Europe, with anthropophilic dermatophytes, such as *Microsporum audouinii, Trichophyton tonsurans* and *Trichophyton soudanense*, becoming increasingly frequent as well [17]. In terms of Tinea pedis and cruris, *Trichophyton rubrum* (anthropophilic) is the most frequently isolated cause [18,19]. However, regarding zoophilic dermatophytes specifically, *Trichophyton mentagrophytes* is most commonly diagnosed [20]. Children under 5 years of age, adults older than 65 years of age, the immunocompromised, and pregnant women are all at an increased risk of contracting zoonotic diseases [17,21]. Those that work in veterinary and human medicine are also at an increased risk, as they are more likely to come into direct contact with those infected [22]. A study conducted in the United Kingdom (UK) found this disease was the zoonosis most frequently contracted by members of the veterinary profession with 58.6% of individuals reporting a confirmed or suspected case [23]. Another study of the veterinary profession in Australia reports similar findings and states that dermatophytosis was the zoonosis most frequently contracted (45.1% of individuals reporting a confirmed case) [24].

Considering the wide range of species affected by dermatophytosis, their highly contagious and infectious nature, the ease of transmission and its zoonotic potential, it is essential for both medical and veterinary practitioners to know what to do when presented with a suspected case. There is also evidence to suggest significant underestimation of the number of cases in both humans and animals, along with increasing infections from new and/or emerging species [25]. There are additional pressures to avoid zoonotic transmission, such as the increasing resistance to commonly used antifungal treatments in both animals and humans [26]. This information should ideally be given in the form of evidence-based clinical guidelines to facilitate rapid integration into clinical decision making.

### 1.2. Evidence based medicine and clinical guidelines

Using evidence-based medicine improves patient outcome and reduces mistakes, risks, and cost, making it an integral part of both human and veterinary medicine decision making [27,28]. Clinical guidelines are methodically developed evidence-based statements that are used by both human and veterinary professionals when making clinical decisions on an individual patient basis [29–32]. They are created with the goal of improving health care. In human medicine, there are strict methodological guidelines to follow during their development. A number of tools have been created for guideline development and appraisal to ensure that only suitably synthesised guidelines are used in practice (e.g., GRADE, AGREE II, GIN-McMaster) [29,33,34]. Even though structured frameworks exist for guideline development in human healthcare, there is still variability in the quality of the guidelines created, and there is a lack of research identifying whether these structured frameworks have been used in veterinary medicine. It is unknown whether evidence-based guidance exists for the identification, treatment, and management of zoonotic dermatophytosis in either animals or humans, and given the zoophilic nature of this disease, if the guidance is reciprocal in its content or extent which warrants further investigation using a scoping review to identify what is published and if there are any knowledge gaps. In order to facilitate a One Health approach to manage this disease, it is key for both veterinary and human healthcare professionals to be united in their advice and recommendations.

The aim of this scoping review was to locate and evaluate all current evidence-based clinical guidelines for zoonotic dermatophytosis in both veterinary and human medicine. Additionally, to determine if consensus between recommendations exists and the presence of synergies between veterinary and human medical advice for the management and prevention of the zoonotic aspects of disease.

## 2. Materials and methods

### 2.1. Protocol and registration

This scoping review was written using the guidelines set by the Preferred Reporting Items of Systematic Reviews and Meta-analysis-Scoping review extension (PRISMA-ScR; [35]). The 22 reporting items listed were consulted by the primary review group (M Brennan – veterinary surgeon and Associate Professor at the University of Nottingham, C O’Connor – final year veterinary student at the University of Nottingham) to ensure a complete and transparent systematic reporting process [36]. The final protocol for this review was not registered. This project received ethical approval from the Committee for Animal Research and Ethics, School of Veterinary Medicine and Science, University of Nottingham (approval numbers UG2021−9 and UG2021−10).

Alongside the primary review group, input was provided for the searching strategy by an information specialist (A Ashmore) and a veterinary student (D Hollister). Assistance was provided for the assessment of the human medicine related papers by two human dermatologists (R Barlow, H Wainman) and a veterinary student (D Hollister). In addition, a panel of experts was consulted across the execution of the review, particularly regarding the search strategy and study design (K Griffiths, L Morrow, J Stavisky, C Kuhl).

### 2.2. Eligibility criteria

This review was not limited to the English language and no date restrictions were imposed. Google Translate and DeepL Translate were used to translate non-English results [37,38]. In addition, papers had to be accessible either via the University of Nottingham or the British Library. If they could not be accessed by the review group, they were excluded.

The search was limited to specific dermatophytes, namely the most common zoophilic species found in humans according to Moriello, Coyner (1) as *Microsporum canis, Trichophyton mentagrophytes (including Arthroderma vanbreuseghemii), and Trichophyton verrucosum.* These species were selected as they relate to canines, felines, and bovines, as well as humans, and are most likely to be mentioned in guidelines discussing this zoonotic infection. A pilot search was conducted to assess whether guinea pigs or other small mammals should be included, but no papers were returned and therefore search terms relating to these species were not included (e.g., *Arthroderma benhamiae*). No other restrictions were imposed.

### 2.3. Information sources

An initial search was run in October 2021 and was repeated in June 2024 to ensure that the results remained up to date as the review was written.

Two separate searches were conducted each time, one seeking veterinary medicine guidelines and the other seeking human medicine guidelines. Medline and Embase were used for both the veterinary and human guidelines search, with the addition of CAB Abstracts for the veterinary guidelines search [39]; all were on the OVID platform.

To supplement the literature database searches and to locate additional guidelines, a targeted grey literature search was conducted. Sources were chosen based on likelihood to contain guidance on dermatophytosis (S1 Table 1 in S1 File).

### 2.4. Search

The extended review group (C O’Connor, D Hollister, M Brennan, L Morrow, J Stavisky, C Kuhl, K Griffiths) drafted a search strategy and list of search terms with assistance from an information specialist (A Ashmore). The search terms centred around a combination of terms relating to ‘dermatophytosis’, ‘guidelines’ and veterinary species of interest (cats, dogs, cows) for the veterinary searches, and terms relating to ‘dermatophytosis’ and ‘guidelines’ for the human medicine searches.

For each veterinary medicine database search, each group of terms were searched individually, with no filters applied, and then combined with ‘AND’ (S1 Table 2 in S1 File). For each human medicine database search, each group of terms were searched individually, with the ‘human’ filter applied, and then combined with ‘AND’ (S1 Table 3 in S1 File).

All resulting papers were imported to Endnote. Duplicates were removed using the automated duplicate removal function, followed by a manual duplicate removal process.

Search terms within each of the grey literature resources were less specific, with information being sought on dermatophytosis in general, with more specific screening criteria applied afterwards.

### 2.5. Selection of sources of evidence

Titles and abstracts of all papers were assessed first. A shortlist of included papers was generated, and the full text of these papers was screened (Tables 1 and 2). The same screening process and criteria were applied to sources identified in the grey literature search.

**Table 1:** Exclusion/inclusion criteria for title and abstract, and full text, screening applied in the scoping review of zoonotic dermatophytosis clinical guidelines in veterinary medicine.

Title and abstract screening criteria	
Papers were included if they:	Papers were excluded if they:
1. Mentioned ringworm, dermatophytosis, dermatophytes, or named specific zoophilic dermatophyte species, e.g., *Microsporum canis, Trichophyton mentagrophytes, Arthroderma vanbreuseghemii* and *Trichophyton verrucosum.* 2. Mentioned zoonoses, zoonosis, zoonotic disease, or discussed transmission from specific animal species including cattle, dogs or cats specifically in the context of dermatophytosis. 3. Mentioned guidelines or protocols.	1. Mentioned dermatophyte species outside of the ones of interest. 2. Did not mention zoonoses, zoonosis, zoonotic disease or transmission from specific animal species (e.g., cattle, dogs or cats) specifically in the context of dermatophytosis. 3. Were focused on dermatophytosis from animal species besides cattle, dogs and cats. 4. Did not mention guidelines or protocols.
**Full text screening criteria**	
Papers were included if they:	Papers were excluded if they:
1. Mentioned dermatophytosis caused by the zoophilic dermatophyte species of interest, e.g., *Microsporum canis, Trichophyton mentagrophytes, Arthroderma vanbreuseghemii* and *Trichophyton verrucosum.* 2. Mentions zoonotic disease, zoonoses, zoonosis or discussed transmission of disease from animals specifically in the context of dermatophytosis. 3. Describes at least some detail as to the risk factors for zoonotic transmission, e.g., at-risk groups, likely transmission routes etc. 4. Were focused on dermatophytosis from animal species including cattle, dogs, and cats. 5. Mentioned guidelines or protocols for dermatophytosis. 6. Were published in a peer-reviewed journal (as determined by the University of Nottingham’s NUsearch function, or Google).	1. Did not discuss dermatophytosis caused by the zoophilic dermatophyte species of interest. 2. Did not discuss zoonotic disease, zoonoses, zoonosis or discuss transfer of disease from animals to humans specifically in the context of dermatophytosis. 3. Did not provide detail as to the risk factors for zoonotic transmission. 4. Were focused on dermatophytosis from other animal species besides cattle, dogs, and cats. 5. Did not provide guidelines or protocols for dermatophytosis. 6. Were not peer-reviewed.

**Table 2:** Exclusion/inclusion criteria for titles and abstract, and full text, screening applied in the scoping review of zoonotic dermatophytosis clinical guidelines in human medicine.

Title and abstract screening criteria	
Papers were included if they:	Papers were excluded if they:
*1.* Mentioned ringworm, dermatophytosis, dermatophytes, or named specific zoophilic dermatophyte species, e.g., *Microsporum canis, Trichophyton mentagrophytes, Arthroderma vanbreuseghemii* and *Trichophyton verrucosum.* 2. Mentioned clinical manifestation of dermatophytosis (e.g., tinea capitis). 3. Mentioned guidelines or protocols.	1. Did not mention ringworm, dermatophytosis, dermatophytes, or named specific zoophilic dermatophyte species. 2. Did not mention clinical manifestation of dermatophytosis (e.g., tinea capitis). 3. Did not mention guidelines or protocols.
**Full text screening criteria**	
Papers were included if they:	Papers were excluded if they:
1. Discussed zoonotic disease, zoonosis, zoonoses or transfer of disease from animals specifically in the context of dermatophytosis. 2. Described at least some detail as to the risk factors for zoonotic transmission, e.g., at-risk groups, likely transmission routes etc. 3. Discussed dermatophytosis caused by the zoophilic dermatophyte species of interest, e.g., *Microsporum canis, Trichophyton mentagrophytes, Arthroderma vanbreuseghemii* and *Trichophyton verrucosum.* 4. Provided guidelines or protocols for dermatophytosis. 5. Were published in a peer-reviewed journal (as determined by the University of Nottingham’s NUsearch function, or Google).	1. Did not discuss zoonotic disease, zoonoses, zoonosis or transfer of disease from animals to humans specifically in the context of dermatophytosis. 2. Did not describe details of the risk factors for zoonotic transmission, e.g., at-risk groups, likely transmission routes etc. 3. Did not discuss dermatophytosis caused by the zoophilic dermatophyte species of interest. 4. Did not provide guidelines or protocols for dermatophytosis. Were not published in a peer-reviewed journal (as determined by the University of Nottingham’s NUsearch function, or Google).

The entire literature search and screening process was done in duplicate. During each search, at least two of three possible reviewers (M Brennan, D Hollister, C O’Connor) conducted the searches and title and abstract screening independently and then discussed their findings. Any discrepancies between assessments were discussed and if agreement could not be reached, a third reviewer was consulted. A shortlist of included papers was generated, and the full texts of these papers were screened independently by two of the same three reviewers with the same stipulations for the disagreements process. This resulted in a final list of papers for data extraction.

For the grey literature searches, the initial screening was done in duplicate (D Hollister and C O’Connor), and repeat searches were done independently by a primary reviewer (C O’Connor).

### 2.6. Variables assessed and charting process

A data charting form was designed to extract data from the included papers (S1 Table 4 in S1 File). The charting variables used were dermatophyte species discussed, animal species discussed, prevalence and risk factors, zoonotic risk factors, zoonotic recommendations for humans, transmission, diagnostic testing, treatment, monitoring response to treatment, and prevention and management.

Data extraction of veterinary papers was completed by two authors (M Brennan, C O’Connor). Data extraction of human papers was completed by two external human medicine dermatologists (R Barlow, H Wainman) as well as an author who had performed data extraction on the veterinary papers (C O’Connor) to ensure extraction methods were consistent for both groups of guidelines.

Data extraction results were compared across papers to determine if there were synergies between the recommendations, with particular interest being paid to the zoonotic aspects of the information.

### 2.7. Critical appraisal of individual sources of evidence

Critical appraisal of the papers was conducted using the criteria in the AGREE II Tool [29].

### 2.8. Synthesis of results

A narrative synthesis of results was undertaken, with comparison between the human medicine and veterinary papers, specifically in relation to the zoonotic aspects of the disease.

## 3. Results

### 3.1. Selection of sources of evidence

**Fig 1:**
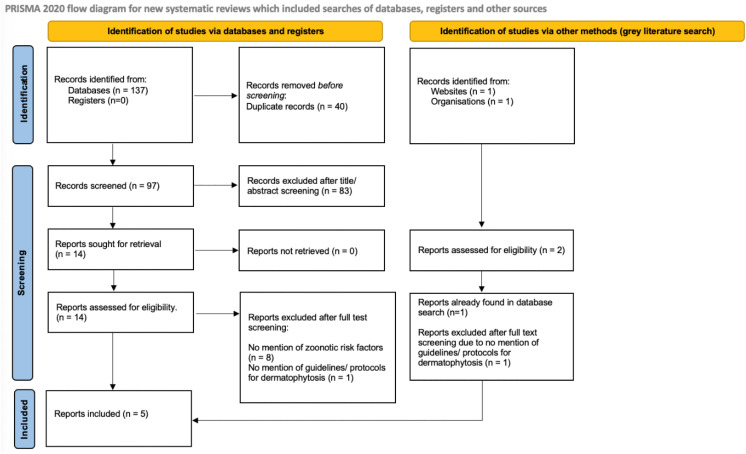
PRISMA Flow Chart for scoping review of zoonotic dermatophytosis clinical guidelines in veterinary medicine [36].

**Fig 2:**
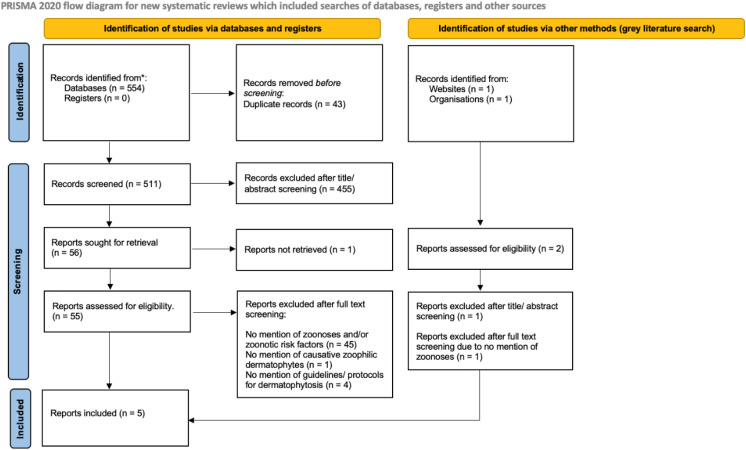
PRISMA Flow Chart for scoping review of zoonotic dermatophytosis clinical guidelines in human medicine [36].

The process and results have been illustrated in two PRISMA flow charts (Figs 1 and 2). For veterinary guidelines, 137 papers were identified in the database search (Fig 1). Forty of these were removed as duplicates, leaving 97 to be screened. Of these 97 papers, 83 were excluded during the title and abstract screening process because they did not meet the inclusion criteria, with 14 papers going forward to full text screening. Five of the 14 papers met all the inclusion criteria and underwent data extraction. Regarding the grey literature search, two papers were located with one being excluded as it had already been found in the database search, and the other being excluded as there was no mention of guidelines/ protocols for dermatophytosis.

For human guidelines, 554 papers were identified in the database search (Fig 2). Forty-three of these were removed as duplicates, leaving 511 to be screened. Of these 511 papers, 455 were excluded during the title and abstract screening process because they did not meet the inclusion criteria, and 56 papers underwent full text screening. Of these 56 papers, one could not be accessed and was therefore excluded, and five were found to have met all the inclusion criteria and underwent data extraction. Regarding the grey literature search, two papers were found, with one being excluded during title and abstract screening, and the other after full text screening due to no mention of zoonoses. In total, 10 papers were eligible for full review. For both the veterinary and human guideline screening phases, there was 100% agreement between reviewers, therefore a third reviewer was not required.

### 3.2. Characteristics of sources of evidence

The types (or characteristics) of papers reviewed varied from summarising what other authors had written, to a literature review, to descriptions of expert opinion gathering. None of the evidence sources that met the review inclusion criteria could be classified as evidence-based guidelines (i.e., none were developed using a specific purposeful framework such as GRADE or AGREE).

The origin, aims, design, method, evidence discussed, and variables discussed are highlighted in Tables 3 and 4. The sample size of many veterinary studies was small. For example, Moriello, Coyner (1) reference 10 studies discussing miconazole + /- chlorhexidine as a topical treatment, with the maximum number of participants reaching 32. This is contrasted by human studies referenced by Mayser, Nenoff (40) regarding itraconazole treatment that had 100 and 167 participants respectively.

**Table 3:** Characteristics of papers included in a scoping review of zoonotic dermatophytosis clinical guidelines in veterinary medicine.

Veterinary papers	Cabañes [40]	Frymus et al. [4]	Moriello et al. [1]	Peano et al. [41]	Schnieder [42]
Origin	Revista iberoamericana de micología (Ibero-American Journal of Mycology)	The Advisory Board of Cat Diseases, published in the Journal of Feline Medicine and Surgery.	World Association for Veterinary Dermatology.	Department of Animal Production, Epidemiology and Ecology, University of Turin with information adapted from the European Scientific Counsel Companion Animal Parasites.	Journal of Consumer Protection and Food Safety, with information adapted from the European Scientific Counsel Companion Animal Parasites.
Aims	To provide information on the new guidelines for dermatophytosis from the World Association of Veterinary Dermatology.	To provide guidelines on prevention and management of dermatophytosis in cats including transmission, clinical signs, diagnosis, treatment, and management.	Investigate existing recommendations and provide updated guidelines for veterinary staff as well as the public on dermatophytosis in dogs and cats.	To provide information on dermatophytosis (and other fungal infections) including management and control in a way that is comprehensible to the reader.	To provide information on dermatophytosis in dogs and cats including diagnosis, treatment, and prevention.
Design	Summary of previously published guidelines.	In depth review of previously published literature with specific focus on cats.	In depth review of all previously published literature on dermatophytosis in dogs and cats (with other animals mentioned).	Adaptation of ESCCAP guidelines on superficial mycoses (including dermatophytosis) published in 2011.	Adaptation of ESCCAP guidelines on dermatophytosis published in 2009.
Method	None written.	None written.	The authors formed a guideline panel and assessed all literature published before 2016. A literature review was completed, and a consensus was reached on most suitable recommendations. The World Association of Veterinary Dermatology supervised this review.	The ESCCAP guidelines are used in combination with information/ anecdotes from the authors, as well as assessing other existing literature. No method is given for this literature search.	Stated that the recommendations are based on current scientific information, but no method is given to say how the literature was found.
Evidence discussed	No studies are discussed in detail. Evidence-based medicine not discussed.	The authors discuss evidence-based medicine in this paper, and grade three studies on a scale of 1–4 (Mostl, 2009). Other studies are discussed but not graded. Not all studies are described in detail, but some are named as ‘controlled’ and ‘placebo-controlled double-blind’ to help determine trustability. Authors highlight that some areas are lacking in studies.	Studies discussed are: in vivo studies, co-habitant exposure studies, laboratory studies, field studies, experimental studies, in vitro studies, peer-reviewed studies, controlled studies, survey studies. Authors state that varying methodologies makes comparison between studies difficult. Sample size varies in each study. Evidence based medicine is discussed but individual studies are not rated.	Studies discussed: in vitro studies, in vivo studies, retrospective studies, controlled studies. Lack of evidence is discussed in terms of vaccine efficacy. No further information is given; evidence-based medicine not discussed.	‘well-controlled’ studies are discussed in terms of human medicine. No further information given; evidence-based medicine not discussed.
Variables discussed	Variables included in these sources include transmission, zoonotic risk, dermatophyte species of importance, animal species of importance, diagnosis, treatment, management, and prevention. Some sources discuss all variables, with others only discussing a few.				

**Table 4:** Characteristics of papers included in a scoping review of zoonotic dermatophytosis clinical guidelines in human medicine.

Human papers	Czaika and Zuberbier [43]	Drake et al. [44]	Drerup and Brasch [45]	Mayser et al. [46]	Seebacher et al. [47]
Origin	Die Dermatologie (The Dermatologist)	Journal of the American Academy of Dermatology.	Paediatrics Monthly.	Journal der Deutschen Dermatologischen Gesellschaft. (Journal of the German Dermatological Society)	Mycoses.
Aims	To answer the question of whether there is a consensus agreement between national and international guidelines to recommend combination therapy for dermatomycoses (including dermatophytosis) or if different recommendations have been made.	This document was created to assist medical professionals as well as others outside the profession in the care of individuals infected with dermatophytosis.	Tinea capitis is increasing in incidence in Germany and this paper was created to help avoid misdiagnoses and poor treatment protocols. Information is also given on prevention of re-infection and contagion.	These updated guidelines were formed to assist medical practitioners with the diagnosis and treatment of those with tinea capitis.	Not explicitly stated but it is clear that these guidelines were formed to provide information on clinical signs, diagnosis, treatment, and prevention of tinea capitis to medical professionals.
Design	Answering a specific question regarding combination therapy recommendations.	This is one of six reports formed using the most up to date data at the time, which summarises recommendations for dermatophytosis.	Summary of current guidelines regarding tinea capitis in children specifically.	Guideline document formed by a multidisciplinary committee regarding tinea capitis.	Guideline document formed by three German medical societies.
Method	A detailed literature review was completed, with the method described in detail in the paper.	None written.	None written.	A literature review was completed, with special attention being paid to the 2010 European Society for Paediatric Dermatology guidelines and the 2014 British Association of Dermatologists guidelines. Further details are given in the document. The Division of Evidence-based Medicine assisted with forming the methodology.	The Expert Group of the German-speaking Mycology Society, the German Dermatology Society, the Professional Association of German Dermatologists, and the German Hospital Hygiene Society worked together to form this paper, but no other information is given.
Evidence discussed	For each guideline discussed, level of evidence is discussed and so is the grade of recommendation and whether literature is directly associated with recommendations given.	Studies and evidence-based medicine is not discussed in detail.	Studies are listed, but not in detail. There is no mention of evidence-based medicine.	Studies discussed: retrospective studies, comparative studies. When providing recommendations, a ‘strength’ is awarded for interpretation ranging from strong to not recommended. This is based off of evidence available. The authors highlight where evidence is lacking. The Division of Evidence-based Medicine provided methodological assistance for this paper.	Studies discussed: Interventional studies, meta-analysis of all studies, dose range studies. Authors discuss a lack of studies in certain areas. Evidence-based medicine is not discussed.
Variables discussed	Variables included in these sources include transmission, zoonotic risk, dermatophyte species of importance, animal species of importance, diagnosis, treatment, management, and prevention. Some sources discuss all variables, with others only discussing a few.				

### 3.3. Critical appraisal of included papers

As none of the papers met the guidelines criteria, a targeted critical appraisal process was undertaken using some of the criteria from AGREE II [29]. Three domains were assessed: Stakeholder involvement (domain 2), rigour of development (domain 3), and applicability (domain 5). Each domain contained a list of criteria that was assessed and subsequently awarded a score between 1 and 7, with 7 being strongly agree and 1 being strongly disagree (S1 Table 5 in S1 File). Once scoring was complete, a percentage domain score was calculated. This was done independently by a primary reviewer (C O’Connor) and results were discussed with another researcher (M Brennan) to determine the final outcomes. Results can be seen in Table 5 and 6. Regarding veterinary guidelines, the paper written by Moriello, Coyner (1) had the highest domain scores across those assessed when compared to the other 4 papers. The target population had been identified but their views had not been sought, a method was described but it was incomplete (search terms and a full search strategy not given), and no information was given on how the guidance should be updated.

**Table 5:** A targeted critical appraisal assessment used during a scoping review of zoonotic dermatophytosis clinical guidelines in veterinary medicine [29].

	Domain Score		
Domain 2 – Stakeholder Involvement	Domain 3 – Rigour of Development	Domain 5 – Applicability
Cabañes [40]	5%	4%	0%
Frymus et al. [4]	11%	23%	37%
Moriello et al. [1]	56%	58%	45%
Peano et al. [41]	55%	29%	42%
Schnieder [42]	50%	23%	45%

**Table 6:** A targeted critical appraisal assessment used during a scoping review of zoonotic dermatophytosis clinical guidelines in human medicine [29].

	Domain Score		
Domain 2 – Stakeholder Involvement	Domain 3 – Rigour of Development	Domain 5 – Applicability
Czaika and Zuberbier [43]	28%	60%	25%
Drake et al. [44]	44%	4%	17%
Drerup and Brasch [45]	44%	27%	46%
Mayser et al. [46]	50%	27%	50%
Seebacher et al. [47]	22%	19%	33%

Regarding human guidelines, the paper written by Mayser, Nenoff (40) had the highest domain scores when compared to the other 4. Key information missing was aspects of stakeholder descriptions (e.g., their discipline, their role in guideline development), views of the target population, and a description of the methodology.

### 3.4. Data extracted from individual sources of evidence

The data extracted from the veterinary and human medicine guidance (including a summary of the veterinary and human medicine papers) about dermatophytosis can be seen in S1 Table 6 and S1 Table 7 in S1 File. The guidance across disciplines appears to be limited in specific areas, particularly those relating to disease prevalence, transmission, and prevention, with more of a focus on diagnosis and treatment. The veterinary guidance discussed all charting variables with varying detail. The majority of human papers also discussed all variables, with the paper by Czaika and Zuberbier (44) not discussing diagnostic testing, monitoring response to treatment, or prevention and management or zoonotic recommendations. Animal species mentioned related to cats and dogs only.

### 3.5. Zoonotic transmission

To directly compare zoonotic recommendations across all papers, data were extracted that specifically related to zoonotic disease (Table 7). While there were some synergies between veterinary and human guidance, there were some gaps identified, with less literature found regarding disease prevalence and the characterisation of transmission between animals and humans.

**Table 7:** Zoonotic recommendations extracted from papers found during a scoping review of zoonotic dermatophytosis clinical guidelines in veterinary and human medicine.

	Cabañes [40]	Frymus et al. [4]	Moriello et al. [1]	Peano et al. [41]	Schnieder [42]	Czaika and Zuberbier [43]	Drake et al. [44]	Drerup and Brasch [45]	Mayser et al. [46]	Seebacher et al. [47]
Advise seeking medical advice (for owners/ animal keepers)	No	No	No	No	Yes, advises that owners should be referred to a medical practitioner if their pet is infected.	No	No	Yes, advises contacting the health department if many people are affected (zoonotic risk can be implied but is not explicitly stated).	Yes, advises that whole families must be seen/ screened in certain cases.	Yes, advises notifying the public health department so that epidemiological exams and prevention rules can be decided on (zoonotic risk can be implied but is not explicitly stated).
Advise seeking veterinary advice (for owners/ animal keepers)	No	No	No	Yes, advises regular testing and immediate treatment of infected pets.	Yes, advised to treat infections adequately.	No	Yes, advises treating animals when needed	Yes, advises seeking vet advice for infected pets	Yes, advises seeking vet advice for infected pets	Yes, advises having pets treated/ examined by their vet
Advise wearing appropriate PPE	No	Yes, gloves are suggested.	No	No	No	No	Yes, but not in the context of zoonotic infection	No	No	Yes, PPE is recommended when handling infected material
At risk groups for zoonotic infection identified	Yes, specific groups are listed.	No	Yes, specific groups are listed.	Yes, specific groups are listed.	Yes, specific groups are listed.	No	Yes, specific groups are listed.	Yes, specific groups are listed.	Yes, specific groups are listed.	Yes, specific groups are listed.
Additional zoonotic advice	None	Improve hygiene including prompt disinfection of animal related injury.	Educate families on handling infected pets.	Improve personal hygiene and limit contact with infected animals. Improve education of those at risk, i.e., vets, breeders, cattery staff. Advises doctors and vets to work together to manage this zoonotic disease.	Improve personal hygiene and limit contact with infected animals. Communicate advice to owners, vets, animal keepers, family members etc.	Infected pets can be the source of infection (especially imported exotic pets).	Avoid contact with infected animals.	Pets can be the source of infection.	Pets can be the source of infection.	Pets can be the source of infection.

Greyed cells indicate the information was present in the paper.

Most papers listed specific groups of people most at risk for zoonotic infection, including those who are immunosuppressed, pregnant, younger than 5 years of age, and older than 65 years of age. Some job roles were identified as carrying higher risk, for example veterinarians and zookeepers, but only by a minority of papers. In addition, most papers listed at least one zoonotic recommendation, with the main suggestion relating to avoiding infected animals. One veterinary paper suggested contacting a human medical practitioner and another advised veterinary and human practitioners to work together, but the information is brief and lacks detail. Two human papers explicitly state that veterinarians should be sought for advice regarding infected pets, with two other papers implying that veterinarians should be contacted by stating that infected pets should be treated. However, this information was stated briefly with minimal supporting information. Two human papers discussed notifying the Public Health Department and another mentions the possibility of whole families requiring screening for infection in certain cases, but it is not stated whether this is due to zoonotic concern or otherwise. Important recommendations such as using personal protective equipment (PPE) to protect from zoonotic spread and to improve education of those at risk are only recommended by 2 out of 10 papers. Additional recommendations include improving personal hygiene, but no information is provided on how to do this appropriately and stating that infected pets are a source of disease, with minimal information on how to prevent infection.

No information was given in the papers in relation to educating owners of infected animals, despite dermatophytosis being extremely contagious.

## 4. Discussion

### 4.1. Key findings

While the ten veterinary and human guidance papers found in this scoping review did meet the inclusion criteria of ‘discussing guidelines or protocols for dermatophytosis,’ none of them could be classed formally as a clinical guideline according to frameworks such as AGREE II or GRADE [29,33]. Recommendations are provided, but none of the papers used a structured published methodology for guideline construction, which highlights a research gap in this area. Although some of the consensus recommendations provide substantial detail [1,46,43], the lack of methodological description makes it difficult to understand how conclusions have been drawn.

It is clear that certain areas are lacking in sufficient research in both veterinary and human medicine, namely prevalence of disease and the description of transmission between animals and humans. For example, dermatophytosis uncommonly affects dogs and cats but the zoonotic risk is high due to close contact with humans; comparatively dermatophytosis are common in cattle, and zoonoses are common in in-contact individuals (e.g., farm workers) but not the general population because of a lack of contact. Limited research is also noted in human medicine. Czaika and Zuberbier (44) were unable to find sufficient literature to support certain treatment protocols for not only dermatophytosis, but other fungal infections as well. It has been reported that the lack of studies on dermatophytosis could be due to its non-fatal and non-reportable status, even though it is a common condition in humans and other animals [1], or possibly because dermatophytosis can resolve spontaneously, which is a management approach frequently used in cattle. The implications of this lack of research for clinical practice in either veterinary or human medicine are numerous. It could limit the standard of care animals and humans receive and likely increase the risk of zoonotic transmission between animals and humans as a result. A lack of knowledge about the at-risk human groups (e.g., veterinarians – particularly those working with cattle, farm workers) most likely to be presenting to medical practitioners with dermatophytosis means an increased rate of misdiagnosis in these human patients. Both types of clinician are left to use other, less robust sources of evidence for decision-making which could also result in more varied care which has repercussions on the length of time it takes for cases to resolve, and potentially transmission to a larger number of animals and/or people. Prolonged care also has financial implications within both veterinary and medical systems, and may increase the risk of development of resistance to therapy.

There appears to be minimal synergy between veterinary and human zoonotic recommendations in many papers, despite dermatophytosis being one of the most common zoonotic dermatological diseases [7–9]. The majority of papers list at-risk groups for zoonotic infection, but very little additional information was provided. In addition, at-risk groups focused mainly on children and those who are immunocompromised, with few papers expanding to include farmers, veterinarians, and other animal handlers who are at a higher risk than other parts of the population because of frequency of exposure. There is also little mentioned about petting zoos and the risks they pose. Some of these differences between veterinary and human zoonotic recommendations may be as a result of differing contexts, such as different environmental factors influencing diagnosis, management and treatment of disease [9]. There is little detailed information on what owners should do if they suspect their pet to be infected and recommendations for how to prevent zoonotic infection, including specifics of effective environmental and hygiene measures, are vague. The papers also do not describe what an infected animal looks like, and therefore owners/animal keepers may not know what clinical signs to look out for. Animal owners/keepers should also be made aware that animals can carry disease asymptomatically (e.g., cats).

There are several areas that are lacking in supporting evidence in both veterinary and human medicine and require further research to be conducted to assist with the development of more evidence-based guidelines. In particular, more research is required regarding the zoonotic aspects of dermatophytosis, disease transmission, and prevalence. Zoonotic risk is discussed by all papers found in this scoping review, but the information provided was minimal. There are no detailed protocols for humans to follow in the case of suspected transmission from animals and considering transmission rates are unknown and dermatophytosis is often under-reported, it is important that further research is undertaken in this area [1]. This may be particularly pertinent as some animals (e.g., cats) can be asymptomatic carriers so there is a need to establish if human infections originate from likely sources (e.g., pets rather than other types of animal).

In order to adopt a more robust One Health approach to this zoonosis and create guidelines that are beneficial to both veterinary and human professionals, these studies must consider both veterinary and human aspects of the infection, as well as any impacts on the environment (e.g., development of antifungal resistance because of medicines ending up in the environment [48]). The environmental impact of veterinary parasiticide treatments has come into question recently for the veterinary profession [49] so closer scrutiny on the environmental impacts of veterinary products generally is likely in the future. It is documented that some One Health approaches are primarily focused on the human health perspective [50,51]. Significant problems with underreporting in both animal and human cases, and the fact that the condition does not often result in catastrophic outcomes (e.g., death) means that leveraging funding for research from both animal and human funders is unlikely. A lack of avenues for interdisciplinary research funding has been reported previously and is likely to be the case here [52], although the recent COVID-19 pandemic has resulted in some interdisciplinary funding calls [53]. However, the nature of these types of interdisciplinary funding reflect exactly the reasons previously highlighted for a lack of joined up thinking such as that most national or global efforts focusing on the preparedness and response to re-emerging and existing infectious diseases, instead of moving into a more preventative focus on zoonotic diseases and environmental-based health risks [54]. A lack of centralised funding flexible enough to be used where it is needed [55] has also been a criticism. New thinking is required to avoid similar situations in the future.

Given that no correctly synthesised guidelines were found in this scoping review, a clear set of evidence-based guidelines for dermatophytosis in both veterinary and human medicine should be created. Researchers will need to determine what information is missing, particularly methodologies, zoonotic information, and instructions on how recommendations can be implemented in practice. The relevance of producing a single set of guidelines for both medical and veterinary practitioners would be to promote a One Health approach to dermatophytosis, with the aim that medical doctors consider a potential zoonotic infection at the initial presentation of dermatophytosis, particularly in individuals at increased risk due their lifestyle or occupation, as well as those who are immunologically compromised. This requires more high quality information on transmission risk, both directly from infected individuals and indirectly from the environment, across a range of situations (e.g., pet owners, farmers, veterinarians, children visiting petting zoos, animal shelter employees). Veterinarians can advise on the most appropriate investigative strategy in cases where zoonotic infection is suspected. This is particularly challenging due to the potential for asymptomatic carriage and infections in animals, possible exposure to multiple animals, and the limitations of testing (including the absence of a ‘gold standard’ test and financial constraints). More high quality information is needed to develop pragmatic, yet effective, strategies across a range of scenarios. An effective treatment regime can be implemented for infected individuals, both animal and human, and in-contacts where necessary. This would optimise time to clinical resolution, promote the responsible use of antifungal agents and minimise the risk of further infection spread. However, this is a challenging area as guidelines would need to consider treatment efficacy, practicality, safety, licensing and cost-effectiveness, which will vary across the range of host species and environments. Advice can be given by medical doctors and veterinarians on specific hygiene measures that should be taken to minimise the risk of disease transmission, both within and across host species, including zoonotic spread. This may require further investigations to establish the most effective, safe and feasible measures, recognising that these may vary with the clinical situation (e.g., vacuuming is not feasible in a farm environment, but the use of antifungal environmental disinfectants may be limited in the home).

The results of this study show that there is no one source of information for practitioners to use when presented with a case of dermatophytosis and this may contribute to variation in standards of care due to the use of different resources.

### 4.2. Limitations

Only two databases were searched for human guideline papers and three for veterinary papers which opens the possibility that some papers might have been missed. This could have implications for the conclusions that can be drawn from this work. Part of the reason for this decision was due to project time constraints. In ideal circumstances, more time would have been available for the review. However, the study design decisions made were done purposively and with consideration of how best to limit the impact of these constraints. The databases chosen were those that are shown to contain the most human and veterinary related research [39,56]. In addition, these databases were selected with the assistance of an advising librarian with extensive experience in this area, with additional mitigations by the grey literature search and backward citation searching.

Secondly, the search strategy was very specific. Any papers found that did not discuss guidelines or protocols in the title or abstract were excluded which could have resulted in missed relevant papers. However, it was hoped that by selecting on this basis, the searches would identify guidelines that were created using robust methodologies. Specific animal and dermatophyte species were selectively searched for, meaning that guidelines relating to other species of animal or dermatophyte could have been missed. However, the species selected were the most frequently identified as causes of zoonotic dermatophytosis in the literature and advice was sought from an experienced librarian when drafting the search strategy.

Google Translate and Deep L were used to translate non-English papers. During the screening process, it is possible that some information in papers was not interpreted correctly, thus affecting inclusions and exclusions. However, the translation software was primarily used during title and abstract screening. Only a few non-English papers made it through initial screening to full text assessment, and inclusion eligibility was able to be determined, and data easily extracted.

An appraisal against all domains within AGREE II was not undertaken on the final set of papers identified as a result of this review. This was purposively done as none of the included set of citations found were created using evidence-based guideline methodologies (e.g., GRADE). A selection of domains from the AGREE II tool were assessed to demonstrate the methodological limitations of the work that has been published, but were not intended to be all encompassing. It is possible that by not assessing the papers against all of the AGREE II domains, important features of the research that exists on this topic may not have been sufficiently represented.

## 5. Conclusion

This scoping review has identified a lack of evidence-based guidelines available to both human and veterinary practitioners regarding dermatophytosis. Additionally, in the literature studied, there is sometimes little synergy between human and veterinary advice in relation to the zoonotic aspect of disease and is likely to result in substandard, inconsistent care being given to both animal and human patients. This highlights the need for One Health guidelines that encompass both human and veterinary medicine perspectives to ensure consistent care is given to both animal and human patients. Ideally cross-discipline collaboration is required in the construction of these guidelines. If this can be achieved, unnecessary zoonotic transmission events will be avoided and standards of care improved for both human and animal patients.

## Supporting information

S1 File Further details of the search strategies used, data charting forms applied and more comprehensive details of content found within the literature found in a scoping review of zoonotic dermatophytosis clinical guidelines in veterinary and human medicine. (DOCX)

S2 File Completed PRISMA reporting checklist. (DOCX)
