# Supplementary material for: A comparison of the existing recommendations for human and veterinary clinicians on the management and prevention of the zoonotic aspects of dermatophytosis: A scoping review
Source: PLoS One. 2026 Mar 12;21(3):e0344010. doi: 10.1371/journal.pone.0344010 (PMC12981445; doi:10.1371/journal.pone.0344010)
Supplement: S1 File — (DOCX) [file pone.0344010.s001.docx]

**S1 Table 1. Grey literature sources for scoping review of zoonotic dermatophytosis clinical guidelines in veterinary and human medicine**

| **Source** | **Access** |
| --- | --- |
| RCVS Knowledge | <https://knowledge.rcvs.org.uk/quality-improvement/tools-and-resources/guidelines/> |
| The World Small Animal Veterinary Association | <https://wsava.org/global-guidelines/> |
| The World Association for Veterinary Dermatology | <https://wavd.org/> |
| Clinician’s Brief | <https://www.cliniciansbrief.com/> |
| Vet Stream | <https://vetstream.ltd/> |
| The National Animal Disease Information Service | <https://www.nadis.org.uk/> |
| The British Association of Dermatologists | <https://www.bad.org.uk/guidelines-and-standards/> |
| The University of Nottingham’s Centre of Evidence-Based Dermatology | <https://www.nottingham.ac.uk/research/groups/cebd/index.aspx> |
| The National Health Service | <https://www.nhs.uk/> |
| The Primary Care Dermatology Society | <https://www.pcds.org.uk/> |
| The Guidelines International Network | <https://g-i-n.net/> |
| The ECRI Guidelines Trust | <https://guidelines.ecri.org/> |
| Medical and Nursing Clinical Key | <https://www.clinicalkey.com/nursing/#!/> |
| Cochrane | <https://www.cochrane.org/> |

**S1 Table 2:** **Literature databases and search terms used for the identification and retrieval of zoonotic dermatophytosis clinical guidelines in veterinary medicine**

|  | Medline | Embase | CAB Abstracts |
| --- | --- | --- | --- |
| Search Terms | 1. (ringworm OR dermatophytosis OR dermatophytoses OR epidermophytosis OR epidermophytoses OR dermatomycosis OR dermatomycoses OR tinea OR tineas OR trichophyton verrucosum OR trychophyton verrucosum OR trichophyton mentagrophytes OR microsporum canis OR arthroderma vanbreuseghemii OR arthroderma benhamiae).mp. OR exp Tinea/ OR exp *Dermatomycoses/ OR exp *Trichophyton/ OR exp *Microspora/ OR exp *Arthrodermataceae/   AND   1. (practice guideline* OR clinical guideline* or guideline*).mp. OR exp *Guideline/ OR exp Practice Guideline/   AND   1. (cat OR cats OR feline OR felines OR felis OR Felidae).mp. OR exp Cats/ OR exp Felis/ OR exp Felidae/ OR   (dog OR dogs OR canine OR canines OR canis).mp. OR exp Dogs/ OR  (cow OR cows OR cattle OR bovine OR bovines OR bovis OR Bovidae).mp. OR exp Cattle/ | 1. (ringworm OR dermatophytosis OR dermatophytoses OR epidermophytosis OR epidermophytoses OR dermatomycosis OR dermatomycoses OR tinea OR tineas OR trichophyton verrucosum OR trychophyton verrucosum OR trichophyton mentagrophytes OR microsporum canis OR arthroderma vanbreuseghemii OR arthroderma benhamiae).mp. OR exp tinea/ OR exp *dermatophytosis/ OR exp *dermatomycosis/ OR exp *Trichophyton verrucosum/ OR exp *Trichophyton mentagrophytes/ OR exp *Arthroderma benhamiae/   AND   1. (clinical guideline* OR practice guideline* OR guideline*).mp. OR exp practice guideline/   AND   1. (cat OR cats OR feline OR felines OR felis OR Felidae).mp. OR exp cat/ OR exp Felidae/ OR   (dog OR dogs OR canine OR canines OR canis).mp. OR exp dog/ OR exp Canis/ OR  (cow OR cows OR cattle OR bovine OR bovines OR bovis OR Bovidae).mp. OR exp cow/ OR exp bovine/ | 1. ­Ringworm.mp. OR dermatophytosis.mp. OR dermatophytoses.mp. OR dermatomycosis.mp. OR dermatomycoses.mp. OR tinea.mp. OR tineas.mp. OR trichophyton verrucosum.mp. OR trychophyton verrucosum.mp. OR trichophyton mentagrophytes.mp. OR microsporum canis.mp. OR arthroderma vanbreuseghemii.mp. OR arthroderma benhamiae.mp. OR exp Trichophyton verrucosum/ OR exp Trichophyton mentagrophytes/ OR Microsporum canis/ OR exp Arthroderma benhamiae/ OR exp Arthroderma vanbreuseghemii/   AND   1. (practice guideline* OR clinical guideline* OR guideline*).mp. OR exp guidelines/   AND   1. (cat or cats or feline or felines or felis).mp. OR exp cats/ OR felidae.mp. or exp Felidae/ OR   (dog or dogs or canine or canines or canis).mp. OR exp dogs/ OR exp Canis/ OR  (cow or cows or cattle or bovine or bovines or bovis).mp. OR exp cows/ OR exp Bovidae/ |

**S1 Table 3:** **Literature databases and search terms used for the identification and retrieval of zoonotic dermatophytosis clinical guidelines in human medicine**

|  | Medline | Embase |
| --- | --- | --- |
| Search terms | 1. (ringworm OR dermatophytosis OR dermatophytoses OR epidermophytosis OR epidermophytoses OR dermatomycosis OR dermatomycoses OR tinea OR tineas OR trichophyton OR trychophyton OR microsporum OR arthroderma).mp. OR exp Tinea/ OR exp *Dermatomycoses/ OR exp *Trichophyton/ OR exp *Microsporum/ OR exp *Arthrodermataceae/   AND   1. (practice guideline* OR clinical guideline*).mp. OR exp practice guideline/   Used ‘human’ filter | 1. (ringworm OR dermatophytosis OR dermatophytoses OR epidermophytosis OR epidermophytoses OR dermatomycosis OR dermatomycoses OR tinea OR tineas OR trichophyton OR trychophyton OR microsporum OR arthroderma).mp. OR exp tinea/ OR exp *dermatophytosis/ OR exp *dermatomycoses/ OR exp *Trichophyton/ OR exp *Microsporum/ OR exp *Arthoderma/   AND   1. (clinical guideline* OR practice guideline*).mp. OR exp practice guideline/   Used ‘human’ filter |

**S1 Table 4 Data Charting Form for scoping review of zoonotic dermatophytosis clinical guidelines in veterinary and human medicine**

|  | **Paper 1** | **Paper 2** | **Paper 3** | **Paper 4** | **Paper 5** |
| --- | --- | --- | --- | --- | --- |
| Dermatophyte species discussed |  |  |  |  |  |
| Animal species discussed |  |  |  |  |  |
| Prevalence and risk factors |  |  |  |  |  |
| Zoonotic risk factors |  |  |  |  |  |
| Zoonotic recommendations for humans |  |  |  |  |  |
| Transmission |  |  |  |  |  |
| Diagnostic testing |  |  |  |  |  |
| Treatment |  |  |  |  |  |
| Monitoring response to treatment |  |  |  |  |  |
| Prevention and management |  |  |  |  |  |

**S1 Table 5: A targeted critical appraisal tool, with specific domains taken from AGREE II [1] that was used to critically appraise each of the included studies.**

| **Domain name** |  | **Strongly Disagree - 1** | **2** | **3** | **4** | **5** | **6** | **Strongly Agree - 7** |
| --- | --- | --- | --- | --- | --- | --- | --- | --- |
| **Domain 2: Stakeholder involvement** | The guideline development group includes individuals from all relevant professional groups |  |  |  |  |  |  |  |
|  | The views and preferences of the target population (patients, public etc.) have been sought |  |  |  |  |  |  |  |
|  | The target users of the guideline are clearly defined |  |  |  |  |  |  |  |
|  | **Total across criteria** |  |  |  |  |  |  |  |
|  | **Percentage domain score (/21)** |  |  |  |  |  |  |  |
| **Domain 3: Rigour of development** | Systematic methods were used to search for evidence |  |  |  |  |  |  |  |
|  | The criteria for selecting the evidence are clearly described |  |  |  |  |  |  |  |
|  | The strengths and limitations of the body of evidence are clearly described |  |  |  |  |  |  |  |
|  | The methods for formulating the recommendations are clearly described |  |  |  |  |  |  |  |
|  | The health benefits, side effects, and risks have been considered in formulating the recommendations |  |  |  |  |  |  |  |
|  | There is an explicit link between the recommendations and the supporting evidence |  |  |  |  |  |  |  |
|  | The guideline has been externally reviewed by experts prior to its publication |  |  |  |  |  |  |  |
|  | A procedure for updating the guideline is provided |  |  |  |  |  |  |  |
|  | **Total across criteria** |  |  |  |  |  |  |  |
|  | **Percentage domain score (/56)** |  |  |  |  |  |  |  |
| **Domain 5: Applicability** | The guideline describes facilitators and barriers to its application |  |  |  |  |  |  |  |
|  | The guideline provides advice and/or tools on how the recommendations can be put into place |  |  |  |  |  |  |  |
|  | The potential resource implications of applying the recommendations have been considered |  |  |  |  |  |  |  |
|  | The guideline presents monitoring and/or auditing criteria |  |  |  |  |  |  |  |
|  | **Total across criteria** |  |  |  |  |  |  |  |
|  | **Percentage domain score (/28)** |  |  |  |  |  |  |  |

**S1 Table 6: Completed data charting form for the scoping review of zoonotic dermatophytosis clinical guidelines in veterinary medicine**

|  | Cabañes [2] | Frymus et al. [3] | Moriello et al. [4] | Peano et al. [5] | Schnieder [6] |
| --- | --- | --- | --- | --- | --- |
| Dermatophyte species discussed | Microsporum canis is the most common zoophilic dermatophyte seen.  The Trichophyton genera is also discussed. | Microsporum canis is the most common.  Other causative dermatophytes include Microsporum gypseum (not zoophilic), Trichophyton mentagrophytes, Trichophyton quinckeanum, and Trichophyton verrucosum. | In the human patient, the most common dermatophyte species seen was Trichophyton rubrum, which is not zoophilic.  Other dermatophyte species: Microsporum canis (cats, dogs, humans), Microsporum equinum (horse), M. persicolor (voles), M. nanum (pig), Trichophyton equinum (horse), T. verrucosum (cattle, humans), Arthroderma benhamiae and vanbeuseghemii and other species of T. mentragrophytes (rodents, rabbits, hedgehogs). | Microsporum canis (most common), Microsporum persicolour, Microsporum gypseum, Tricophyton mentagrophytes, Trichophyton erinaceid, Trichophyton rubrum, and arthroderma benhamiae. | Microsporum canis, M gypseum, M persicolor, and Trichophyton mentagrophytes. |
| Animal species discussed | Microsporum canis is most commonly found in cats and dogs.  Cats are the main reservoir for Microsporum canis. | Most information is given regarding cats.  Dogs are discussed briefly. | Majority of studies discussed in this paper used feline and canine species.  Other animals discussed are horses, voles, pigs, cattle, rodents, rabbits, and hedgehogs. | Information is given regarding dogs and cats.  Other animals discussed are horses, small rodents including guinea pigs, rabbits, hamsters, foxes, camels, and hedgehogs. | T mentagrophytes is listed as the most common pathogen in guinea pigs, rats, and other small animals.  M canis is listed as the most common in cats and dogs. |
| Prevalence and risk factors | The true prevalence of dermatophytosis is challenging to ascertain, as it is not a reportable disease.  Young animals are at a higher risk of contracting the disease.  Microsporum canis is the most common cause isolated in dogs and cats (usually over 90% isolation). | Dermatophytosis is the most common fungal infection and one of the most important infectious skin diseases in cats.  Prevalence of subclinical infection is quite low.  Higher prevalence in FIV and FeLV infected cats has not been confirmed.  Cats less than two years of age, immunosuppressed, infected with other diseases, and those with nutritional deficiencies are at higher risk.  High temperatures and humidity, moving new cats into a shelter environment, attending cat shows, skin trauma, poor hygiene, overcrowding leading to social stress, and boarding in catteries are also important risk factors.  Outdoor cats can be exposed via digging to M gypseum (not zoophilic), via small rodents to T mentagrophytes or quinckeanum, and via cattle with T verrucosum. | Prevalence ranges from 0.5-3.6% worldwide in different studies.  Warmer climates, e.g. countries in South America, have a higher frequency of cases.  Young animals and strays are more at risk.  Lifestyle is also a risk factor, with working dogs more likely to be exposed to fungal spores and contract disease (German Short-haired Pointers, Fox Terriers, Labrador Retrievers, Belgian Groenendael, Beagles, Pointers, Jack Russell Terriers, German Shepherd Dogs and Jagdterriers).  Cats with FIV and FeLV are not at an increased risk of contracting infection.  Determining prevalence and breed disposition is challenging because dermatophytosis is not a spontaneously occurring disease, is not a notifiable disease, and it is not a fatal disease.  Persian cats are commonly reported to have the infection. | Working dogs and outdoor cats are at an increased risk of contracting T mentagrophytes and occasionally M canis.  Lactating animals can pass infection to their young.  Puppies and older animals are at an increased risk.  Persian cats, Dalmatians, Poodles, Jack Russell Terriers, Manchester Terriers, and Yorkshire Terriers are predisposed.  Higher prevalence in FIV/ FeLV infected cats has not been proven.  Ectoparasite infections i.e. Cheyletiella can cause skin microtraumas leading to dermatophyte infections.  High temperatures and humidity are also predisposing factors, as is attending animal competitions and too frequent bathing with inappropriate products leading to micro-trauma which increases the risk of infection. | Increased risk in younger animals and in those immunosuppressed.  Lactating animals can pass infection to their young.  All breeds are susceptible, but Persian cats have been listed as predisposed.  Other predispositions include familial relations in cats, ectoparasites and secondary infections causing pruritus (lead to microtrauma),and any disease that causes immunosuppression.  Warm and humid conditions, as well as breeding/ shelter environments + attending competitions also increase risk.  Prevalence is likely to be higher in countries with larger stray animals. |
| Zoonotic risk factors | Dermatophytosis can occur in any individual, but children less than 5 years of age, adults over 65 years of age, pregnant women, and those who are immunocompromised are at an increased risk of contracting the disease. | Handling infected animals can increase the risk of zoonotic transmission (contact with spores). | Those most at risk from contracting zoonotic disease are children under 5 years of age, adults older than 65 years of age, pregnant women, and the immunocompromised.  Direct contact with hair or skin lesions of the infected, and also fomites in the environment.  Immunocompromised human patients are discussed, and those who have had an organ transplant, those with CARD9 deficiency, and those with HIV are more likely to present with severe dermatophytosis. | Catteries pose a high risk.  Children and immunocompromised people are also at high risk.  Others at risk are those on immune suppressive therapies i.e. organ transplants, and those with HIV  The source of most M canis infections is an infected cat. | Contact with infected animals and the environment.  Those that are elderly, pregnant, infected with HIV, undergoing chemotherapy, have had an organ transplant, or are being treated for an autoimmune disease are at an increased risk.  In addition to vets, those working in shelters or farms are also at an increased risk.  It is also listed that children or those with mental disabilities might display a behaviour that puts them at risk of infection (i.e. not able to understand risk). |
| Zoonotic recommendations for humans | Apart from stating that this infectious disease can be both treated and cured, no other information is given. | Humans should undertake special hygienic measures when handling potentially infected animals. This includes using gloves and prompt disinfection of animal related injuries such as scratches. | The most frequent complication seen in immunocompromised patients with M canis infections is an extended treatment period.  It is possible to treat and cure dermatophytosis.  Family members (especially children) should be advised about risk of infection from handling infected animals. | Good personal hygiene.  Regular testing and immediate treatment in pets.  Reduce contact with infected animals and environment.  Educate people on risks of contacting infected animals (especially vets, dog/cattery workers, breeders, etc) | Good hygiene.  Treat existing infections adequately.  Avoid contact with infected animals.  Reduce exposure of children and other at-risk people to contaminants.  Adequate education for pet owners about risks.  If a pet and an owner both have dermatophytosis, the owner should be referred to a medical practitioner; should be close cooperation between veterinarians and doctors. |
| Transmission | An accurate rate of transmission from animals to humans is unknown due to a lack of studies in this area.  Dermatophytosis is transmitted via direct contact with fur or skin lesions of infected individuals (both animal and human).  Transmission is also possible via indirect contact with fomites contaminated with scales and hair from infected individuals. | Dermatophytosis is transmitted via direct contact with infected animals (or carrier/ subclinical animals) and indirect contact with fomites including brushes, dust particles, and clothing. | No studies have been conducted into the spread of disease from a pet to a healthy owner, and therefore an accurate rate of transmission is unknown.  Contracting disease due to environmental contamination alone is not common.  Transmission occurs via direct or indirect contact +/- concurrent trauma to skin. | Transmission occurs via direct contact (symptomatic or asymptomatic carrier) or through fomites (i.e. shoes, clothes, brushes, towels, and environmental contamination).  Spores can remain viable for up to 18 months in optimal humidity and temperature. | Infectious material consists of hairs, blankets, collars, spores in the environment, and infected animals. |
| Diagnostic testing | There is no gold standard diagnostic test.  Diagnosis and confirmation of dermatophyte species involved can be achieved via culture, which is highly sensitive and can identify the species involved. Culture is also used to identify mechanical carriers and asymptomatic individuals.  Direct microscopy is also done, a test which is less sensitive but highly specific.  PCR can also be used but may provide a false positive result as the test can detect DNA from non – viable dermatophytes. | Wood’s lamp is a simple and relatively cheap method of diagnosis although it is not very sensitive (only 50% of M canis strains fluoresce) and false positives are possible. This test should be confirmed using an alternative diagnostic method.  Other diagnostic methods include direct microscopy (pluck hairs from edge of lesion or with Wood’s lamp guidance) which is also not very sensitive and can give false positives, fungal culture on sabouraud dextrose agar (gold standard with high sensitivity and species identification), and PCR for M canis.  It is recommended to obtain the sample for culture using a sterile toothbrush. | There is no gold standard diagnostic test.  Hair with an active infection can be diagnosed using Wood’s lamp and direct microscopy. The authors state that the comment ‘not all strains will fluoresce’ is not true.  Dermatophyte species can be diagnosed via fungal culture using the toothbrush technique and Sabouraud’s dextrose agar.  Dermoscopy can be used to locate hairs for culture and direct examination.  PCR is possible but can cause false positives due to nonviable DNA.  In scenarios where there are unusual or nodular lesions, a biopsy can be performed for diagnosis. | Wood’s lamp is a good screening test in cats and dogs.  Direct microscopy of hair is another choice, but a good level of knowledge is required.  Fungal culture is gold standard, and hair can be collected with a toothbrush or other collection methods.  Colony counting can be used to distinguish between carriers and infected animals, but this technique is still developing, and results do not always correlate. | Wood’s lamp is step one in the protocol described.  If results are negative but you have a high index of suspicion, then trichograms are done for direct microscopy.  If still negative and suspicious, then a fungal culture is preformed which is the most reliable method.  Biopsy is also possible. |
| Treatment | For successful treatment, systemic antifungals (for example itraconazole or terbinafine) must be used in combination with a topical disinfectant. | Disease is self-limiting in immunocompetent individuals after 1-3 months.  For successful treatment, systemic and topical treatment must be used in combination for at least 10 weeks/ until no lesions are seen, and dermatophytes cannot be cultured on 2 coat brushings with 1-3 weeks between.  Itraconazole is the preferred systemic antifungal (unless pregnant), with terbinafine being another option. Ketoconazole and griseofulvin have too many side effects. Lufenuron has not been proven to have anti-fungal effects in cats.  Regarding topical treatment, it is recommended to rinse cats with either enilconazole solution or miconazole (with or without chlorhexidine), as spot treatment has a limited efficacy. Lime sulphur is also an option.  Topical treatment alone is less effective due to poor penetration and lack of tolerance.  It is also recommended to decontaminate the environment with lime sulphur, enilconazole, or diluted household chlorine bleach.  In feline patients with a limited number of lesions, it is recommended to gently clip (to avoid microtrauma) the hair, providing a wide margin around the lesions.  For feline patients with generalised lesions, those that are longhaired breeds, and those entering a cattery, it is beneficial to clip the entire cat to aid application of topical therapy (i.e. better penetration)  An efficacious vaccine is not available for the feline patient (are believed to reduce lesion size). | Topical Treatment: the most effective therapies recommended are shampoos containing lime sulphur, miconazole in combination with chlorhexidine, or enilconazole. These are applied bi-weekly.  Accelerated hydrogen peroxide products, Climbazole and terbinafine shampoos are not definitively recommended.  Systemic Therapy: The most effective therapies are itraconazole and terbinafine. griseofulvin is also effective but is more likely to cause adverse reactions.  Ketoconazole and fluconazole are less effective and lufenuron is not effective at all.  Vaccines do not prevent infection but could potentially be used as a therapy in combination with other treatments.  To successfully treat dermatophytosis, systemic and topical therapy must be used together. | This is a self-limiting disease, and spontaneous recovery is possible in healthy animals.  Treatment is used to shorten the duration of infection and limit contagion.  Systemic medication is used to accelerate recovery and topical medication is used to reduce transmission and contamination of the environment.  Itraconazole is the ideal systemic therapy (minimal side effects in comparison to others – ketoconazole, griseofulvin, terbinafine, lufenuron).  Topical therapies include shampoos (imidazoles + chlorhexidine, miconazole) and dermatological solutions (enilconazole, miconazole, lime sulphur.  Shaving the coat (gently) is also recommended despite no controlled studies to prove this. It allows better penetration of topical medication.  Pharmacological resistance is rare. | In most cases, the infection is self-limiting in immunocompetent animals.  Treatment is given to reduce duration and prevent contagion.  Topical treatment is given to minimise transmission/ contamination and systemic treatment is given to speed recovery.  Topical treatment should be applied to the whole animal and should be applied bi-weekly. Options include enilconazole or miconazole shampoo.  Systemic treatment options are itraconazole (first choice), griseofulvin, or terbinafine.  Clipping the coat is also recommended, as it improves drug penetration for topical therapy.  Resistant dermatophytes have only been proven in minimal cases. |
| Monitoring response to treatment | Culture can be used to monitor response to treatment. | Culture can be used to monitor response to treatment.  Treatment must be carried on until fungal culture results are negative. The test must be carried out twice, the second being 1-3 weeks after the first. | A negative PCR test is indicative of cure in a treated cat.  Additionally, Wood’s lamp, fungal cultures, and monitoring clinical signs can be utilised to assess response to treatment. | Sample the animal once a month during treatment. Treatment can be stopped once two negative cultures are found. | Treatment is applied until therapeutic success (2 negative results, 4 weeks apart). The first culture should be taken 4 weeks after the start of treatment.  It should be noted that 3 negative tests are required in some circumstances i.e. shelter animals.  If therapy is not successful, you should consider owner compliance, any underlying disease, and hereditary predispositions. |
| Prevention and management | Little information is provided on management apart from treatment advice.  No information is given on prevention. | Owner compliance and a good treatment programme are essential in treating dermatophytosis (especially in catteries and shelters) to prevent spread and reinfection.  Arthrospores are susceptible to high temperatures, as well as lime sulphur, enilconazole, or household bleach.  Ringworm is rarely recurrent but can be difficult to eradicate in catteries and shelters.  Separate infected and non-infected animals with extensive decontamination of the environment. All animals in the cattery should be treated, or divide the cats into groups and treat according to infection status. | Environmental decontamination (i.e. with household bleach or enilconazole) is recommended in order to prevent and reduce contamination and to also prevent false positive dermatophyte cultures. Other disinfectants are accelerated hydrogen peroxide, potassium peroxymonosulfate and over the counter general disinfectants.  Bedding, carpets, and other household items should be washed and decontaminated as well.  Environmental culture/ sampling is not recommended unless you are worried about false positives.  Contamination can be reduced via coat clipping, topical treatment, and regular cleansing.  It is also possible to confine an animal to prevent spread, but this can have long lasting negative impacts on behaviour. | Environmental decontamination is recommended with an appropriate antifungal (i.e. dilute bleach, 1% formalin, enilconazole).  There are vaccines available in some European countries, but it is not effective in dogs/cats.  New arrivals should be quarantined and undergo a screening test i.e. Wood’s lamp and culture.  Where animals are housed in large numbers, it is important to consider an overall treatment and prevention plan that is suitable, physically and economically, for both the animals and the people working in each specific scenario. Protocols should be put in place to reduce disease occurrence by using isolation, quarantine and decontamination.  People working in high risk environments (e.g. shelter workers) should be made aware of the appropriate protocols and be informed of how the disease is transmitted  When animals are living closely together, or if animals have specific risk factors, co-habiting animals with infected individuals should be screened and treated if needed.  Doctors and veterinarians should be encouraged to cooperate to manage zoonotic cases more thoroughly. | There is a vaccine available in Germany that, while it doesn’t prevent infection, a higher infectious dose is required for infection to take route.  Environmental cleaning should occur once a week during treatment. Everything the animal has contact with should be disinfected with chlorine bleach or enilconazole.  For kennels etc., animals should remain in quarantine until cleared free of disease or treated.  Recommendations and information on dermatophytosis should be communicated to owners, veterinary professionals, animal keepers – so they can also pass on the message to their family members and others who may come into contact with the animal. |

**Summary of veterinary studies:**

There was consensus on a variety of factors, with some discrepancy on others. All agreed that *Microsporum canis* was either the most common, or one of the most common causes of dermatophytosis, with the majority describing the genera *Trichophyton* as important as well. Dogs and cats were the two main animal species referred to due to their involvement in the majority of dermatophytosis studies. An increased prevalence was reported in animals that were young, immunosuppressed, or those with concurrent disease. In addition, a high humidity/ temperature, overcrowding, and certain lifestyles (i.e. working dogs/ outdoor cats) were reported to increase risk as well. Transmission was through both direct and indirect contact, and Peano et al. [5] stated that spores could last up to 18 months in the environment. Many agreed that there was no ‘gold standard’ diagnostic test and suggested beginning diagnostics with a Wood’s lamp assessment or direct microscopy. Fungal culture was reported as a test having high sensitivity, but the longest duration to achieve a result, which was seen as a disadvantage. Biopsy was reported as being reserved for refractory cases or those with unusual lesions. PCR was discussed as highly sensitive but had the potential for false positives due to detection of non-viable DNA. It was recommended that a combination of topical and systemic therapy was employed, with the most common choices including lime sulphur or miconazole (+/- chlorhexidine) and itraconazole, respectively. Monitoring therapy response was reportedly carried out via fungal culture. Treatment duration was not discussed in detail, only that it should continue until mycological cure. Environmental decontamination was also recommended with, for example, household bleach or enilconazole. Appropriate biosecurity and quarantine measures were suggested, and vaccination was described as not effective by some, and as a way to prevent infection by others.

Regarding zoonotic infection in particular, those most at risk were described as young children less than 5, adults over 65, those who were immunosuppressed, and those who were pregnant. Some papers specified also those with organ transplants, HIV, undergoing chemotherapy, or suffering with an autoimmune disease at a higher risk. Veterinary, farm, and shelter staff were predisposed due to their profession. Guidelines for zoonotic infection were limited in these papers, with most recommending good hygiene, appropriate treatment of pets, and improving owner education. Only one paper, written by Schnieder et al. [6] suggested consulting a human medical practitioner.

**S1 Table 7: Completed data charting form for the scoping review of zoonotic dermatophytosis clinical guidelines in human medicine**

|  | Czaida and Zuberbier [7] | Drake et al. [8] | Drerup and Brasch [9] | Mayser et al. [10] | Seebacher et al. [11] |
| --- | --- | --- | --- | --- | --- |
| Dermatophyte species discussed | Trichophyton soudanense and T violaceum are listed as anthropophilic dermatophytes that can cause tinea capitis and tinea corporis.  Regarding zoonotic/ zoophilic dermatophytes, M canis, T mentagrophytes (also called T interdigitale zoophil) and T verrucosum are listed. | Epidermophyton, Microsporum, and Trichophyton are discussed.  T rubrum, T mentagrophytes, T tonsurans, T verrucosum, M canis, M gypseum, and E floccosum in particular. | Trichophyton, Microsporum, Nannizzia are common.  M canis is the most common cause of tinea capitis in Europe.  Other species are M audouinii, tonsurans, violaceum, and soudanense. T verrucosum, mentagrophytes, benhamiae, and rubrum. | Microsporum canis is the dermatophyte most commonly seen.  Other important dermatophytes include include T metagrophytes, T tonsurans, and T verrucosum.  Others mentioned are T violaceum, M audouinii, and Nannizzia. | Microsporum canis is listed as the most common cause of tinea capitis in Central Europe.  Other dermatophytes discussed are M audouinii, T mentagrophytes, T tonsurans, T soudanense, and T violaceum. |
| Animal species discussed | Cats, guinea pigs, calves, hedgehogs, chinchillas. | Just says ‘animals’ in some places, also discusses cats and cattle. | Rodents i.e. mice, cats, dogs, horses, rabbits, and guinea pigs. | Small rodents i.e. guinea pigs, cats, dogs, horses, monkeys, and rabbits. | Cats, guinea pigs, hamsters, rabbits, and cows. |
| Prevalence and risk factors | Dermatophytosis is becoming increasingly common across the globe.  Those involved in long distance travel or sports competitions are at an increased risk of fungal infections. | 10-20% of the population is estimated to be infected with dermatophytosis.  Tinea pedis is the most common infection, occurring in up to 70% of infected adults.  Trichophyton rubrum is the most common dermatophyte seen with non-scalp infections.  Family members and others in contact with the infected are at risk as well. | Children aged 3-7yrs are mainly affected (adults and newborns are rarely affected).  Incidence is increasing in Germany due to, for example, migration from Africa and travel abroad to areas with endemic dermatophytes.  Global incidence is unknown. | T tonsorans is the most common cause of Tinea Capitis in the US and is often seen in 6-10yr old male children.  In adults, the infection is more common in women due to hormonal influence.  Risks include hormonal disorders, immunosuppression, autoinoculation, and being around infected children. | Attending the gym/ school PE classes.  Children are particularly at risk.  Children in Africa are most commonly infected with T sourdanense. |
| Zoonotic risk factors | There is an increased amount of imported exotic pets. | Those who have HIV or are immunocompromised are at an increased risk.  Farm workers, zookeepers, lab workers, and vets are also listed as people at risk. | Infected pets and pets that are asymptomatic carriers.  Rodents are commonly infected and are popular pets, there was also an increased amount of people getting pets during the Covid pandemic.  Field mice spread infection to cats and dogs that hunt them. | Pets that are infected or asymptomatic carriers. | Infected family pets and household items they have touched i.e. beds, toys, etc.  Touching cats when abroad. |
| Zoonotic recommendations for humans | **None** | Avoid contact with infected animals.  Treat animals appropriately when needed. | Seek veterinary advice for their pets. | Seek veterinary advice for infected animals. | Disinfect contaminated household items.  PPE should be worn when handling infected material.  Have pets treated/ examined by their vet. It is recommended that they receive systemic treatment. |
| Transmission | Direct contact with animals and people. | Direct contact with infected people or animals. | Direct and indirect contact. | Direct and indirect contact i.e. with brushes, razors, towels, bedding, hats, toys, etc (fomites). | Indirect contact with car seats, toys, etc.  Direct contact with infected humans and animals. |
| Diagnostic testing | **None for dermatophytes** | Taking a thorough history including checking for concurrent disease.  Direct microscopy with potassium hydroxide preparation (scrape from edge of new lesions or sample material from pustules/ blisters). Stains can be used (Chlorazol Black E or Parker's blue black ink).  Fungal culture on Sabouraud’s glucose agar.  Bacterial culture to rule out secondary infection.  Wood’s lamp exam is another option.  If the infection is not resolving, biopsy is also possible (i.e. more difficult cases).  Routine allergy testing is not appropriate. | Take a thorough history including pet ownership, travel, and infected family members/ friends.  Fungal culture on Sabouraud’s agar.  Wood’s lamp is also useful for M canis infections.  Direct examination is another possible method, via Fluorescence-optical preparation and potassium hydroxide solution preparation.  PCR is another possibility.  It should be noted that bacterial smears can lead to incorrect diagnoses.  All samples should be taken from the edge of an untreated lesion that is new/ as fresh as possible. | Wood’s lamp to detect a yellow-green fluorescence.  Fungal culture on Sabouraud’s agar with samples collected with a massage brush or other swab method. This can take up to 6 weeks to grow and it is recommended to clean the area with alcohol before collecting the sample to reduce bacterial growth.  Direct microscopy is another suitable option. The sample is prepared with potassium hydroxide.  PCR is another suitable choice, and species can be determined via sequencing or using specific probes i.e. ELISA.  If the patient is testing negative, biopsy is indicated for further investigation. | Direct microscopy after preparation with potassium hydroxide.  Fungal culture is another suitable test (using Sabouraud’s glucose agar or Kimmig agar or Mycosel agar).  Wood’s lamp is another useful diagnostic aid.  If still testing negative but suspicion is high, biopsy is indicated. |
| Treatment | Topical and systemic therapies are recommended to be used together.  Topical therapy, if applied quickly and appropriately, can reduce the need of systemic therapy.  If there is a strong inflammatory reaction, corticosteroids can be used (strength depending on inflammation present). Application should be limited to short-term (i.e. no more than 10 days) due to possible side effects. In addition, the corticosteroid should not be too potent as this will reduce antifungal efficacy.  Possible therapies include imidazole derivatives i.e. miconazole which has good coverage for dermatophytes as well as yeast and some gram-positive pathogens. | Topical treatment alone is suitable for some infections i.e. those that are non-inflammatory. Possible products include imidazole derivatives i.e. econazole, allylamines i.e. terbinafine, ciclopirox olamine, benzoic acid, tolnaftate, haloprogin, drying agents, powders, salicylic acid and possibly antibiotics as well.  Topical corticosteroids can be given as well, but only for short term + low potency due to side effects.  Systemic therapy is indicated with inflammatory infections, for those where topical therapy did not work, chronic infections, or for those immunocompromised. Possible options are griseofulvin, ketoconazole. Evolving therapies include terbinafine and itraconazole.  Surgical therapy is not indicated unless for the drainage of lesions. | It is recommended to commence treatment asap (i.e. not to wait for mycology results) unless you are in doubt of the cause of infection (i.e. not dermatophyte related).  Treatment is aimed to provide relief as quickly as possible with minimal side effects, to prevent hair loss, and to prevent further infection.  Treatment includes antifungal shampoos/ other topical products biweekly for 2-4 weeks.  Regarding systemic treatment, terbinafine and itraconazole are commonly used.  Terbinafine is more effective against Trichophyton species compared to Microsporum species, but it is not approved for treatment in children (neither is itraconazole).  Griseofulvin is approved for children, but is not available in Germany.  Treatment is usually 3 weeks for Trichophyton species and 8-12 for Microsporum (with terbinafine). | For systemic therapy, age, weight, and possible interactions with other medications must be considered.  Topical treatment should be used in combination with systemic therapy to reduce spore count/ infectiousness.  Topical therapy i.e. shampoo should be applied biweekly over the course of 2-4 weeks (everywhere, not just lesion location). Therapies include selenium sulfide, ketoconazole, clotrimazole, and ciclopirox.  Systemic therapies include itraconazole (preferred for M canis) or terbinafine (preferred for Trichophyton). Griseofulvin is another option for children, as the others are not approved.  Treatment is carried out for a minimum of 4 weeks for adults and 6-8 for children.  There is not enough evidence to support intermittent treatment.  Trimming/ shaving hair can also help and reduce the time treatment is required.  Corticosteroids (oral or topical) can be used to speed up the healing process. Should not be used long term to stop side effects.  If there is no improvement, you can increase the dose or switch antifungal agent. | Systemic and topical treatment should be used together to reduce infectiousness and duration of treatment time.  Treat until mycological cure is achieved.  There are different treatment methods for children and adults.  Griseofulvin is the only approved treatment for children. Options for adults include terbinafine, itraconazole, and fluconazole.  Minimum treatment time is 4-6 weeks.  Topical treatment should be administered once daily for 1 week. Antifungal shampoos should be applied biweekly.  Trimming/ shaving hair will also help and can be done once per week. |
| Monitoring response to treatment | **None** | Recurrence occurs in up to 70% of cases, often due to poor compliance with treatment or reservoir of infection in nails for example.  Follow up assessments are indicated depending on severity, extent of lesions, and how well the medications are working.  Hepatic, renal, and haematopoietic functions will need to be checked with systemic therapy use. | There is no consensus on monitoring during therapy.  Treatment is carried out until mycological cure. If the culture is positive, treatment is continued for another 2 weeks and then patient is retested.  If a patient is deemed at risk i.e. of developing liver toxicity, then monitoring should begin prior to therapy commencing and up to 2-4w afterwards. | Repeat fungal cultures are required until mycological cure.  If culture is positive, repeat treatment for a further 2 weeks.  PCR can also be used, as it is more sensitive and quicker than culture.  Lab monitoring is required to check liver values i.e. AST and ALT.  Treatment failure can be due to non-compliance, underdosing, resistance to medications, reinfection, and comorbidities. | Repeated fungal cultures must be done until mycological cure. |
| Prevention and management | **None** | Avoid contact with infected animals, soil, and people.  Wear protective footwear in public facilities. | There is a vaccination available for dogs, horses, and cats and while it doesn’t stop transmission, it does reduce severity.  If many people are affected, the health department must be contacted.  Environment must be decontaminated including household items i.e. laundry, toys, brushes, towels, and bedding.  Children can still go to school, only after they commence treatment. A one-week break is suggested for those with anthropophilic dermatophytes. | Do not send infected children to school until the dermatophyte spore-load they carry is reduced.  It might be required to screen the entire family if a child is infected.  Completely disinfect the environment.  Separate or disinfect or dispose of hair/ skin/ and nail care items that are used by the infected individuals from healthy individuals. | Do not share combs, brushed, nail tools, headgear, etc.  PPE should be worn when handling infected material/ people.  Do not send infected children to schools for 1-2 weeks once treatment has started.  It might be necessary to screen the rest of the family as well.  Environment must be disinfected.  The public health department should be notified and they can decide on epidemiological exams and impose other prevention rules. |

**Summary of human medical studies:**

Similar to the veterinary guidance, there was a consensus on a variety of factors, with some discrepancy on others. The majority listed *Microsporum canis* as the most common dermatophyte seen in human infections, with the genera *Trichophyton* and *Nannizzia* also being described as important. Regarding infected animals, cats and dogs were the most discussed, with cattle, small rodents, and horses also being mentioned. The majority of guidelines referred to Tinea Capitis specifically, with infection being most common in children no older than 10. Regarding dermatophytosis in general, it was reported that prevalence was increasing, and those who travelled long distances, attended sports competitions/ went to the gym, were immunosuppressed, or had hormonal disorders were at an increased risk. Transmission reportedly occurred via direct and indirect contact. It was recommended that diagnosis should begin with a full clinical history and move on to either Wood’s lamp assessment or direct microscopy. Bacterial culture was recommended to rule out secondary infections. PCR was discussed as an option, but stated that false positives were a risk. Fungal culture was recommended, but took a long duration to enact. If index of suspicion was high, but the patient tested negative, it was stated that a biopsy was indicated. A combination of topical and systemic therapy was recommended. Far more options were available when compared with veterinary medicines, with the most common being miconazole (topical) and itraconazole or terbinafine (systemic). Corticosteroids were recommended for an additional anti-inflammatory component of treatment. Fungal culture was recommended for monitoring response to therapy, with the addition of assessing hepatic and renal function due to risks of systemic therapies. Treatment was often recommended to be carried out for at least 10 weeks, until mycological cure. Environmental decontamination was recommended (i.e. with dilute bleach) alongside appropriate hygiene, biosecurity, and quarantine measures.

Regarding zoonotic infection in particular, minimal advice was given which consisted of avoiding infected animals, wearing appropriate PPE, and seeking veterinary advice when needed (not all guidelines recommended this).

# Bibliography

1. Brouwers MC, Kho ME, Browman GP, Burgers JS, Cluzeau F, Feder G, et al. AGREE II: advancing guideline development, reporting and evaluation in health care. Canadian Medical Association Journal. 2010;182(18):E839-E42.

2. Cabañes FJ. Ringworm in cats and dogs: new guidelines. Revisto Iberoamericana de Micologia. 2021;38:1-2.

3. Frymus T, Gruffydd-Jones T, Pennisi MG, Addie D, Belák S, Boucraut-Baralon C, et al. Dermatophytosis in cats: ABCD guidelines on prevention and management. Journal of Feline Medicine and Surgery. 2013;15(7):598-604.

4. Moriello KA, Coyner K, Paterson S, Mignon B. Diagnosis and treatment of dermatophytosis in dogs and cats. Clinical Consensus Guidelines of the World Association for Veterinary Dermatology. Veterinary Dermatology. 2017;28(3):266-e68.

5. Peano A, Pasquetti M, Chiavassa E. Superficial mycoses in dogs and cats. [Micosi superficiali del cane e del gatto}. Summa, Animali da Compagnia. 2012;29(4):40-55.

6. Schnieder T. Control of dermatophytosis in dogs and cats. German adaptation of ESCCAP recommendation no. 2, February 2009. Journal fur Verbraucherschutz und Lebensmittelsicherheit. 2009;4:257-64.

7. Czaika V, Zuberbier T. Local combination therapy of inflammatory dermatomycosis: A review of recommendations in national and international guidelines. Der Hautarzt. 2015;66:360-9.

8. Drake LA, Dinehart SM, Farmer ER, Goltz RW, Graham GF, Hordinsky MK, et al. Guidelines of care for superficial mycotic infectionsof the skin: Tinea corporis, tinea cruris, tinea faciei, tinea manuum, and tinea pedis. Journal of the American Academy of Dermatology. 1996;34(2):282-6.

9. Drerup KA, Brasch J. Tinea capitis in children-A diverse disorder. MONATSSCHRIFT KINDERHEILKUNDE. 2022.

10. Mayser P, Nenoff P, Reinel D, Abeck D, Brasch J, Daeschlein G, et al. S1 guidelines: Tinea capitis. JDDG: Journal der Deutschen Dermatologischen Gesellschaft. 2020;18(2):161-79.

11. Seebacher C, Abeck D, Brasch J, Cornely O, Daeschlein G, Effendy I, et al. Tinea capitis: ringworm of the scalp. Mycoses. 2007;50(3).
